# Exposures to Bleach, Peroxide, Disinfectants, Antimalarials, and Ivermectin Reported to the California Poison Control System Before and During the COVID-19 Pandemic, 2015-2021

**DOI:** 10.1177/00333549231201679

**Published:** 2023-11-07

**Authors:** Alice Ghai, Emily Sabour, Raeann Salonga, Raymond Ho, Dorie E. Apollonio

**Affiliations:** 1 School of Pharmacy, University of California San Francisco, San Francisco, CA, USA; 2 California Poison Control System, San Francisco, CA, USA

**Keywords:** bleach, disinfectants, household cleaning products, peroxide, antimalarials, chloroquine, hydroxychloroquine, ivermectin, COVID-19

## Abstract

**Objectives:**

The COVID-19 pandemic led to widespread fear of infection, with many people expanding their use of cleaning products and trying unproven prevention and treatment strategies. We described shifts in reported exposures related to COVID-19 home interventions.

**Methods:**

This study considered suspected toxicity exposures involving household cleaning products (bleach, peroxide, disinfectants), antimalarials (hydroxychloroquine and chloroquine), and ivermectin reported to the California Poison Control System from 2015 through 2021 and assessed trends in exposures by using interrupted time-series analyses.

**Results:**

We found a significant increase in exposures reported to the California Poison Control System related to household cleaning products and ivermectin during the COVID-19 pandemic. As of January 1, 2015, the baseline level of reported exposures to household cleaning products was 707.33 per month and was declining at a rate of 1.71 (95% CI, –2.87 to –0.56) per month through February 29, 2020. In March 2020, an increase of 466.57 (95% CI, 328.08-605.07) reported exposures above baseline occurred, after which exposures to cleaning products decreased at a rate of 23.40 (95% CI, –32.48 to –14.32) per month. The number of reported exposures to antimalarials did not change significantly before or during the pandemic. The number of reported ivermectin exposures before December 2020 was initially stable at 14.50 per month and then increased by 2.05 per month through December 2021.

**Conclusions:**

Our observations suggest that while some dangerous home prevention and treatment efforts resolve over time, further interventions may be needed to reduce the public health effects related to attempts to self-treat COVID-19 with ivermectin.

The onset of the COVID-19 pandemic and increased public awareness of its health risks were accompanied by individual efforts intended to prevent and treat infections.^1^ These efforts were linked to concern that the virus could be detected for several days on surfaces, even though detection of the SARS-CoV-2 virus does not prove its viability.^2^ Efforts included an expanded use of household cleaning products and uses of medications that could be toxic, that were not effective for COVID-19 prevention and treatment, and that were consumed despite public health warnings.^3,4^ Use of these products and medications was attributable in part to widespread misinformation about COVID-19 prevention and treatment.^1^ In the United States, these toxic exposures are typically reported to state poison control systems.^3-5^

Studies in multiple countries found that an increase in the misuse of household cleaning products during the first months of 2020 was associated with the COVID-19 pandemic.^6-8^ During January–March 2020, poison control centers in the United States reported a 20.4% increase in the number of telephone calls about exposure to cleaners and disinfectants,^9^ and the number of telephone calls about unsafe exposure to hand sanitizer among children significantly increased after the first confirmed case of COVID-19 was reported in the United States.^10^ Of the 45 550 telephone calls to US poison control centers related to exposure to bleach and other disinfectants received during January–March 2020, children aged <5 years represented >40% of cases.^11^ In May 2020, a survey involving 502 US adults found that 39% of respondents had engaged in hazardous activities when attempting to prevent COVID-19 transmission, including using bleach to wash groceries, applying bleach and disinfectants to bare skin, and purposefully ingesting these products.^12^

Reports were made of efforts to self-treat COVID-19 exposure or infection by using treatments for other diseases. Beginning in March 2020, claims that hydroxychloroquine and chloroquine could prevent or treat COVID-19 led to their popular use; from January through April 2020, US poison control centers reported a 42% increase in chloroquine and hydroxychloroquine exposures relative to the same period in 2019.^13^ Popular claims that ivermectin could be used to treat COVID-19 appeared in December 2020,^14^ and these were followed in January 2021 by an increase in the number of reports to poison control centers of ivermectin exposures related to attempts to self-treat COVID-19.^4^ Inappropriate use of ivermectin can result in adverse effects such as joint pain, swollen lymph nodes, fever, and, in some cases, liver injury, as well as dangerous neurologic effects, including seizures and tremors.^14^

Despite media reports on COVID-19 home prevention and remedy attempts, limited research has assessed whether spikes have declined or persisted in the number of exposures related to these attempts that were reported to poison control centers,^6-9^ and much of the research assessing these exposures originated from outside the United States.^6-8^ In this study, we analyzed trends in exposures in California for (1) household cleaning products, including bleach, peroxide, and disinfectants, and (2) medications proposed in the media for COVID-19 prevention and treatment that were not approved for this purpose, including antimalarials (hydroxychloroquine and chloroquine) and ivermectin.

## Methods

### Study Design

This retrospective medical record review analyzed all suspected toxicity cases of (1) household cleaning products (eg, bleach, peroxide, and disinfectants) and (2) medications such as hydroxychloroquine, chloroquine, and ivermectin to prevent and treat COVID-19 that were reported to the California Poison Control System (CPCS) before (January 1, 2015, through February 29, 2020) and during (March 1, 2020, through December 31, 2021) the COVID-19 pandemic.

### Data Source and Data Collection

CPCS is a 24-hour hotline dedicated to providing the public and health professionals with advice on potentially toxic exposures and preventive services; it receives approximately 200 000 telephone calls annually. We extracted data on reported exposures involving bleach, peroxide, disinfectant, hydroxychloroquine, chloroquine, or ivermectin. We used generic codes for household cleaning products, antimalarials, and ivermectin from the American Association of Poison Control Centers (unpublished manual, National Poison Data System Coding Users’ Manual Version 4.3, 2020) to identify case records from the CPCS database (eTable 1 in Supplemental Material). CPCS analysts provided deidentified information on cases associated with the products of interest during the study period. We excluded cases from the study if they met ≥1 of the following criteria: caller location outside of California and telephone calls for information that did not refer to a suspected or actual exposure. We counted and analyzed cases involving multiple substances as separate reports. These reports included information on adverse effects, hospitalizations, and death. The University of California San Francisco Institutional Review Board reviewed and approved the study, and its conduct was consistent with applicable federal and state law allowing waiver of informed consent on the grounds that all data were deidentified.

### Measures

We defined an exposure as an “actual or suspected contact with any substance which has been ingested, inhaled, absorbed, applied to, or injected into the body, regardless of toxicity or clinical manifestation.”^15^ We defined and coded total reported exposures as dependent variables.^15^ We defined and coded product types as independent variables. We defined household cleaning products as bleach, peroxides, and disinfectants. The “bleach” category included hypochlorite (liquid and dry) and nonhypochlorite; the “peroxides” category, peroxides and hydrogen peroxides; and the “disinfectants” category, hypochlorite (nonbleach products) and nonhypochlorite (other or unknown). We classified hydroxychloroquine and chloroquine as antimalarials. We classified ivermectin as an anthelmintic. We included demographic characteristics drawn from case records and variables coded by CPCS that indicated the reason for exposure and route of administration (eg, ingestion, inhalation, ocular, dermal, injection). Where data were missing, we retained the cases and excluded the missing values from analysis.

### Statistical Analyses

We began by generating descriptive statistics of exposures reported to CPCS for all products and medications identified in the study. We classified exposures by the following categorical variables: age group (<18 or ≥18 years [children/adolescents or adults]), self-reported sex (male or female), exposure type, primary reason for exposure, and route of exposure. We calculated percentages and SDs for each categorical variable. We also summarized data (mean and SD) for the following continuous variables: age, the number of substances involved in the exposure, and the number of exposures per month. We summarized data for the 2 study periods—before and during the pandemic (January 1, 2015, through February 29, 2020, and March 1, 2020, through December 31, 2021, respectively)—and for the entire study period. Within each category, we conducted *t* tests to assess significant differences between summary values for the periods before and during the pandemic, with *P* < .05 considered significant.

To determine whether the level of exposures changed significantly during the COVID-19 pandemic, we used interrupted time-series analyses (ITSA) to assess the 2 periods: before and during the pandemic. The latter period represented widespread public awareness of the COVID-19 pandemic (eg, the World Health Organization declaring COVID-19 a pandemic in March 2020).^16^ The standard ITSA regression model for a single group follows the following form: *Y_t_* = β_0_ + β_1_*T_t_* + β_2_*X_t_* + β_3_*X_t_T_t_* + ϵ_*t*_, where β_0_ is the intercept, β_1_ is the slope prior to intervention, β_2_ is the change immediately after the intervention, and β_3_ represents the treatment effect of that intervention over time. Because data are reported as monthly number of exposures, any monthly changes (increases or decreases) before and during the pandemic are reported as coefficients from the regression model (β_1_, β_2_, β_3_). We used the “itsa” plugin for Stata 17 (StataCorp LLC) to conduct the analysis, followed by “actest” to test model assumptions, as well as additional sensitivity analyses.^16^ We also used multiple-group analyses to include additional coefficients that represented differences between groups, such as adults and children, as well as categories of cleaning products.^17^

Annual reports from the American Association of Poison Control Centers have consistently shown that, by age group, the majority of telephone calls reporting potentially toxic exposures involve children aged <5 years.^18^ Given these historically high rates of pediatric exposures, we also conducted a multiple-group ITSA that split exposures involving adults from those involving children for household cleaning products and for the medications studied. In the combined analysis, we used March 2020 as the point of intervention to capture any differences across groups that might not have been observed for antimalarials with a later date.

## Results

### Characteristics of Exposures

From January 1, 2015, through December 31, 2021, a total of 75 714 exposures were reported to CPCS relating to the use of household cleaning products, antimalarials, and ivermectin. Household cleaning products consisted of bleach (37 590 exposures), peroxide (13 093 exposures), and disinfectants (7615 exposures); medications were antimalarials (hydroxychloroquine and chloroquine; 765 exposures) and ivermectin (1411 exposures). We excluded 15 240 exposures that did not meet study criteria (eg, calls for information), leaving 60 474 exposures for analysis (Table 1).

**Table 1.** Characteristics of reported exposures to bleach, peroxide, disinfectants, antimalarials, and ivermectin reported to the California Poison Control System, January 1, 2015, through December 31, 2021^a^

**Characteristic**	**Overall (N = 60 474)**	**January 1, 2015, through February 29, 2020 (n = 43 255)**	**March 1, 2020, through December 31, 2021 (n = 17 219)**	***P* value** ^b^
**Categorical variable, % (SD)**				
Age, y				
<18	34.4 (0.5)	36.9 (0.5)	28.2 (0.5)	<.001
≥18	65.6 (0.5)	63.2 (0.5)	71.8 (0.5)	<.001
Sex				
Female	57.1 (0.5)	56.3 (0.5)	58.9 (0.5)	<.001
Male	42.9 (0.5)	43.7 (0.5)	41.1 (0.5)	<.001
Exposure				
Bleach	62.2 (0.5)	63.0 (0.5)	60.0 (0.5)	<.001
Peroxide	21.7 (0.4)	21.8 (0.4)	21.2 (0.4)	.07
Disinfectant	12.6 (0.3)	11.7 (0.3)	14.9 (0.4)	<.001
Antimalarial	1.3 (0.1)	1.3 (0.1)	1.2 (0.1)	.64
Ivermectin	2.3 (0.2)	2.2 (0.2)	2.7 (0.2)	<.001
Primary reason for exposure^c^				
Unintentional	89.1 (0.3)	89.1 (0.3)	89.3 (0.3)	.49
Intentional	6.2 (0.2)	6.6 (0.3)	5.4 (0.2)	<.001
Route of exposure^d^				
Inhalation	19.7 (0.4)	17.8 (0.4)	24.5 (0.4)	<.001
Ingestion	59.8 (0.5)	62.0 (0.5)	54.2 (0.5)	<.001
**Continuous variable, mean (SD)**				
Age, y	27.9 (23.6)	27.2 (23.6)	30.0 (23.3)	<.001
No. of substances in exposure	1.2 (0.5)	1.2 (0.5)	1.2 (0.6)	<.001
No. of exposures per month	719.9	697.7	782.7	—^e^

a Data source: California Poison Control System.

b Comparing January 1, 2015–February 29, 2020, and March 1, 2020–December 31, 2021, per the *t* test, with *P* < .05 considered significant.

c Excludes less common reasons, such as contamination and tampering.

d Excludes less common routes, such as dermal and ocular.

e No comparison made.

During the study period, the number of exposures for all products averaged 697.7 reports per month before the pandemic and 782.7 during the pandemic (Table 1). Overall, 34.4% of total exposures involved children and adolescents (of these, 78.5% were children aged <6 years), 65.6% were adults, and 57.1% were female.

### Exposures to Household Cleaning Products

**Figure 1.**
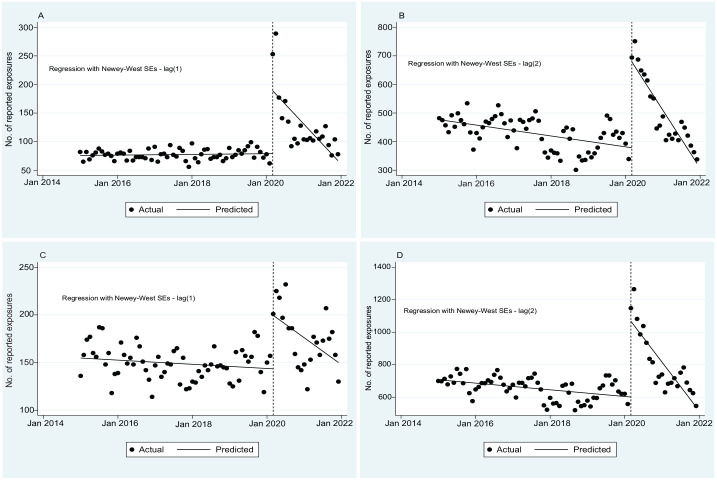
Monthly number of reported and predicted exposures to household cleaning supplies to the California Poison Control System, 2015-2021, before and during the COVID-19 pandemic: (A) disinfectants, (B) bleach, (C) peroxide, and (D) household cleaning products. Data source: California Poison Control System. The vertical dashed line represents the start of the COVID-19 pandemic in March 2020. Note that *y*-axis scales differ for each graph.

From January 2015 through February 2020, the baseline number of monthly exposures to household cleaning products combined (bleach, peroxide, and disinfectants) averaged 707.33 and decreased significantly at a rate of 1.71 (95% CI, –2.87 to –0.56) exposures per month (*P* = .001). In March 2020, this number increased by 466.57 (95% CI, 328.08-605.07) exposures, a 66% increase (*P* < .001). The number of exposures to household cleaning products decreased significantly after March 2020 by an average of 23.40 (95% CI, –32.48 to –14.32) exposures per month (*P* < .001; Figure 1, Table 2).

**Table 2.** Trends in reported exposures to household cleaning products before and during the COVID-19 pandemic generated by interrupted time-series analysis, California, January 1, 2015, through December 31, 2021^a^

	**Estimated monthly exposures, no.**	**Trend in estimated monthly reported exposures (95% CI) [*P* value]** ^b^		
**Cleaning product**	**At baseline, January 1, 2015**	**Before pandemic: January 1, 2015, through February 29, 2020**	**In March 2020**	**During pandemic: March 1, 2020, through December 31, 2021**
All	707.33	−1.71 (−2.87 to –0.56) [.001]	466.57 (328.08 to 605.07) [<.001]	−23.40 (−32.48 to –14.32) [<.001]
Bleach	476.72	−1.58 (−2.47 to 0.70) [.001]	300.50 (231.35 to 369.64) [<.001]	−15.35 (−19.55 to –11.16) [<.001]
Peroxide	154.73	−0.18 (−0.49 to 0.13) [.26]	56.12 (27.68 to 84.56) [<.001]	−2.17 (−4.16 to –0.19) [.03]
Disinfectants	75.88	0.05 (−0.07 to 0.17) [.44]	109.95 (51.12 to 168.78) [<.001]	−5.87 (−9.72 to –2.02) [.003]

a Data source: California Poison Control System.

b Estimates generated by interrupted time-series analysis per the following form: *Y* *t* = β0 + β_1_Tt + β_2_Xt + β_3_XtTt + t, where *Yt* is the aggregated outcome variable measured at each equally spaced time point *t*, *Tt* is the time since the start of the study, *Xt* is a dummy (indicator) variable representing the intervention (preintervention period, 0; otherwise, 1), and *XtTt* is an interaction term. β_0_ represents the intercept or starting level of the outcome variable. β_1_ is the slope or trajectory of the outcome variable until the introduction of the intervention. β_2_ represents the change in the level of the outcome that occurs in the period immediately following the introduction of the intervention (vs the counterfactual). β_3_ represents the difference between pre- and postintervention slopes of the outcome. Significant *P* values in β_2_ indicate an immediate treatment effect, and significant values in β_3_ indicate a treatment effect over time. Within the model, *P* < .05 was considered significant.

### Exposure to Household Cleaning Products Among Adults Versus Children

The baseline number of reported monthly exposures to household cleaning products among adults from 2015 through February 2020 was 414.09 and increased by 392.15 (95% CI, 289.24-495.05) in March 2020, a 95% increase (*P* < .001). In subsequent months, the number of exposures decreased by an average of 19.57 (95% CI, –26.61 to –12.54) per month (*P* < .001). In contrast, among children, the baseline number of monthly exposures from 2015 through February 2020 was 293.24; it increased by 74.43 (95% CI, 47.71-101.15) in March 2020, a 24% increase (*P* < .001), and then decreased by an average of 3.82 (95% CI, –5.41 to –2.24) through December 31, 2021 (*P* < .001; eFigure 1 in Supplemental Material).

### Medication Exposures

**Figure 2.**
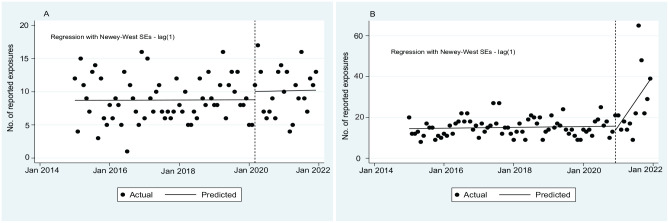
Monthly number of reported and predicted exposures to the California Poison Control System, 2015-2021, before and during the COVID-19 pandemic: (A) antimalarials and (B) ivermectin. Data source: California Poison Control System. The vertical dashed line represents the start of the COVID-19 pandemic in March 2020. Note that y-axis scales differ for each graph.

We found no significant change in the number of reported exposures for antimalarials during the study period (Figure 2, eTable 2 in Supplemental Material). The number of exposures for ivermectin before December 2020 was stable, but after December 2020, it increased by an average of 2.05 (95% CI, 0.58-3.53) per month through December 2021 (*P* = .007).

### Medication Exposures Among Adults Versus Children

Before and after widespread awareness of the pandemic in March 2020, the number of reported exposures for all medications differed significantly between children (16.78 per month before the pandemic and 17.85 during the pandemic) and adults (21.15 and 22.25, respectively; eFigure 2 in Supplemental Material).

## Discussion

Given research from other countries and media reports, we anticipated that the number of reported exposures to household cleaning products and medications that were perceived to prevent or treat COVID-19 would increase beginning in March 2020. As anticipated, the number of exposures to cleaning products and ivermectin increased in California during the COVID-19 pandemic and coincided with the appearance of COVID-19 prevention measures.

Counts of reported exposures to cleaning products, individually and as a category, increased suddenly and significantly in March 2020 but returned to previous levels 12 to 18 months later. Improper use of cleaners and disinfectants can result in adverse health effects, such as skin, eye, respiratory, and gastrointestinal irritation, for individuals using them and for those in close proximity.^12^

We anticipated that the increase in the number of reported exposures to household cleaning products among children would be higher than among adults given (1) the increase in the presence of household cleaning products likely stored in the home and (2) the historical findings that young children may consume them if the products are unsecured.^18^ However, in our sample, adults had higher levels of exposures than children after March 2020. This finding could reflect greater use of household cleaning products in unconventional and unsafe ways by adults, as with the intention to prevent or treat COVID-19. However, overall rates of intentional use represented a small share of cases overall (6%), and intentional exposures during the pandemic were less frequent than they were before the pandemic. Given that most exposures were inadvertent or accidental, we conclude that media reports of extensive purposeful ingestion of household cleaning products were not supported by our data on exposures.

Unintentional exposures among adults could nonetheless be related to media reports proposing ways to prevent COVID-19 transmission. For example, in April 2020, *Consumer Reports* highlighted shortages of household cleaning products, noting that 61% of people in the United States could not find cleaning wipes or disinfectant spray, and 74% said that they had trouble finding hand sanitizer.^19^ In response, some people concocted hand sanitizers and disinfectants using recipes proposed in do-it-yourself YouTube videos.^10^ Most of these videos failed to suggest labeling storage containers, 69% encouraged the use of oils or perfumes to enhance hand sanitizer scent, and 2% promoted the use of coloring agents to make them more attractive to children.^10^ This emphasis on diligent disinfecting practices and the advice to create ad hoc cleaning products during product shortages may have led to increased unintentional exposures.

We analyzed reported exposures for 2 medications that media reports had indicated were misused for COVID-19 prevention and treatment: antimalarials and ivermectin. Media reports in the United States on antimalarial use for the prevention and treatment of COVID-19 appeared in March 2020,^13^ while media reports of ivermectin use for the prevention and treatment of COVID-19 in the United States appeared in December 2020.^14^ Despite high-profile reports of hydroxychloroquine and chloroquine poisonings after intentional use to treat COVID-19,^13^ the number of exposures reported to CPCS remained stable before and during the pandemic. In contrast to household cleaning products and hydroxychloroquine, the number of exposures to ivermectin increased after December 2020 claims that it could be used to prevent or treat COVID-19.^14^ Unlike exposures involving household cleaning products that appeared to return to prepandemic levels, the number of ivermectin exposures continued to increase by 2 per month through December 2021, the end of our study period. We identified no significant differences in the number of exposures between children and adults for medications (hydroxychloroquine, chloroquine, ivermectin).

### Strengths and Limitations

One strength of our study was that we analyzed trends in exposures related to COVID-19 prevention and treatment strategies during a longer period (6 years) than in previous studies,^6-9^ which allowed the identification of time trends before and after widespread popular awareness of the COVID-19 pandemic. Our study also had several limitations. First, exposures in California to the products that we studied were not necessarily reported to CPCS; instead, they were dependent on concern about signs of toxic exposure and public awareness of the existence of poison control centers. Second, exposures not reported to poison control centers were not included in the analysis. Together, these limitations may have resulted in our identification of a higher number of exposures than what actually occurred. Third, our data were specific to a single state, California; as such, they may not be generalizable to trends in other regions. Despite these limitations, these findings may provide new information on how the number of exposures that may have been related to attempts to self-treat with home remedies or prevent COVID-19 transmission with unsafe cleaning practices changed before and during the pandemic. Future research could assess (1) whether these trends in exposures were consistent across regions and during longer periods and (2) the potential differences between children and adults.

## Conclusion

We found that the onset of popular awareness of the COVID-19 pandemic was associated with significant, sudden, and substantial increases in the number of reported exposures to household cleaning products—specifically, bleach, peroxide, and disinfectants—followed by a slow decline. The number of ivermectin exposures, in contrast, increased at an average rate of 2 exposures per month after it became popularized as a COVID-19 treatment in December 2020. For all these exposures, although their number increased among children and adults, they occurred more frequently among adults. Our findings suggest that additional interventions may be needed to address increasing ivermectin exposures, potentially involving increased public health messaging or stricter prescribing guidelines. In addition, clinicians should be aware of these trends and communicate health risks to people receiving care to minimize exposures associated with antimalarials and ivermectin, as well as any potential medications proposed as COVID-19 treatments in the future.

## Supplemental Material

sj-docx-1-phr-10.1177_00333549231201679 – Supplemental material for Exposures to Bleach, Peroxide, Disinfectants, Antimalarials, and Ivermectin Reported to the California Poison Control System Before and During the COVID-19 Pandemic, 2015-2021 Supplemental material, sj-docx-1-phr-10.1177_00333549231201679 for Exposures to Bleach, Peroxide, Disinfectants, Antimalarials, and Ivermectin Reported to the California Poison Control System Before and During the COVID-19 Pandemic, 2015-2021 by Alice Ghai, Emily Sabour, Raeann Salonga, Raymond Ho and Dorie E. Apollonio in Public Health Reports
