# Supplementary material for: Exposures to Bleach, Peroxide, Disinfectants, Antimalarials, and Ivermectin Reported to the California Poison Control System Before and During the COVID-19 Pandemic, 2015-2021
Source: Public Health Rep. 2023 Nov 7;139(1):112–9. doi: 10.1177/00333549231201679 (PMC10905766; doi:10.1177/00333549231201679)
Supplement: sj-docx-1-phr-10.1177_00333549231201679 – Supplemental material for Exposures to Bleach, Peroxide, Disinfectants, Antimalarials, and Ivermectin Reported to the California Poison Control System Before and During the COVID-19 Pandemic, 2015-2021 [file sj-docx-1-phr-10.1177_00333549231201679.docx]

# Associations between the COVID-19 pandemic and exposures to bleach, peroxide, disinfectants, antimalarials, and ivermectin reported to the California Poison Control System (CPCS)

## Figure S1. Reported Cleaning Supply Exposures in Children (<18 years) and Adults (18+ years) Before and During COVID-19 Pandemic


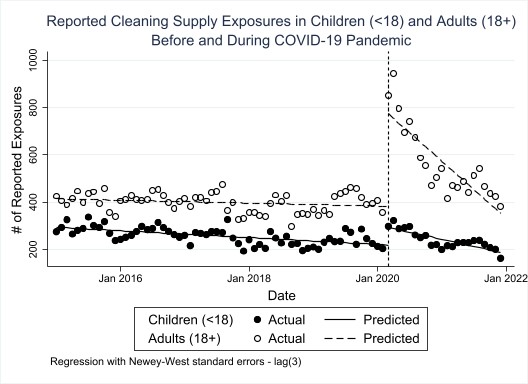


Vertical dashed line represents: March 2020 (start of the COVID-19 pandemic)

## Figure S2. Reported Medication (Hydroxychloroquine, Chloroquine, and Ivermectin) Exposures in Children (<18 years) and Adults (18+ years) Before and During COVID-19 Pandemic


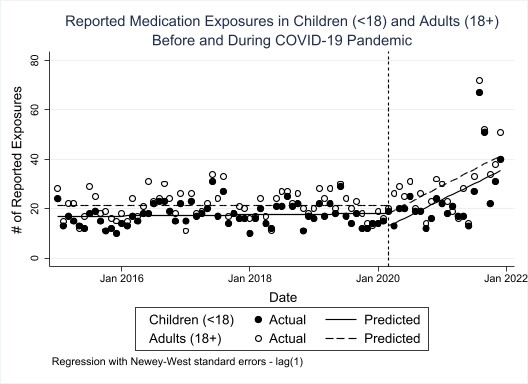


Vertical dashed line represents: March 2020 (start of the COVID-19 pandemic)

## Table S1. AAPCC Product Codes

| 077286 | Disinfectants: other or unknown (Lysol disinfectants) |
| --- | --- |
| 042281 | Disinfectants: Hypochlorite (Non-Bleach Products) |
| 042280 | Bleaches: Hypochlorite (Liquid and Dry) |
| 077280 | Bleaches: Non-Hypochlorite |
| 143320 | Peroxides |
| 143790 | Hydrogen peroxide 3% |
| 077715 | Other types of anthelmintic (including ivermectin, fenbendazole, praziquantel, pyrantel) |
| 077716 | Antimalarials (hydroxychloroquine/chloroquine) |

## Table S2. Trends in Antimalarial and Ivermectin Exposures During COVID-19

|  | Number of exposures in January 2015 | Trend in Exposures from 1 January 2015 to 29 February 2020 | Shift in Exposures in March 2020 for Antimalarials & December 2020 for Ivermectin | Trend in Exposures During COVID-19 Effect |
| --- | --- | --- | --- | --- |
| Antimalarials | 9 | 0.001  [CI: -0.04, 0.05] | 1.25  [CI: -2.80, 5.30] | 0.008  [CI: -0.25, 0.27] |
| Ivermectin | 14 | 0.02  [CI: -0.04,0.07] | -2.06  [CI: -10.55, 6.43] | 2.05**  [CI: 0.58, 3.53] |

CI: 95% Confidence Interval, * p<0.05, ** p<0.01
